# Discovery and antitumor activities of constituents from *Cyrtomium fortumei* (J.) Smith rhizomes

**DOI:** 10.1186/1752-153X-7-24

**Published:** 2013-02-04

**Authors:** Shengjie Yang, Mingchuan Liu, Na Liang, Qi Zhao, Yuping Zhang, Wei Xue, Song Yang

**Affiliations:** 1 State Key Laboratory Breeding Base of Green Pesticide and Agricultural Bioengineering, Key Laboratory of Green Pesticide and Agricultural Bioengineering, Ministry of Education, Guizhou University, Guiyang 550025, P.R. China; 2 Ctr for R&D of Fine Chemicals, Guizhou University, Huaxi St, Guiyang, 550025 China

## Abstract

**Background:**

*Cyrtomium fortumei* (J.) Smith is an important Chinese herbal medicine because of its biological functions. However, systematic and comprehensive studies on the phytochemicals from *Cyrtomium fortumei* (J.) Smith and their bioactivity are limited.

**Results:**

Using the bioassay-guided technique, the ethyl acetate and *n*-BuOH extracts of the rhizomes of *Cyrtomium fortumei* (J.) Smith were shown to exhibit good antitumor activities, consequently leading to the isolation of 23 compounds. All compounds were isolated from the plant for the first time. The inhibitory activities of these compounds were investigated on tumor cells MGC-803, PC3, and A375 *in vitro* by MTT (thiazolyl blue tetrazolium bromide) assay, and the results showed that pimpinellin (3) had potent cytotoxic activities against the three cell lines, with the IC_50_ values of 14.4 ± 0.3 μM, 20.4 ± 0.5 μM, and 29.2 ± 0.6 μM, respectively. The mechanism of the antitumor action indicated that pimpinellin inhibited the growth of MGC-803 cells *via* the induction of tumor cell apoptosis, with apoptosis ratio of 27.44% after 72 h of treatment at 20 μM.

**Conclusions:**

This study suggests that most of the compounds from the roots of *Cyrtomium fortumei* (J.) Smith could inhibit the growth of human carcinoma cells. Moreover, pimpinellin inhibited the growth of tumor cells *via* the induction of tumor cell apoptosis.

## Background

*Cyrtomium fortumei* (J.) Smith belongs to the Dryopteridaceae family which comprises approximately 14 genera and 1700 species throughout the world, and is widely spread in tropical and subtropical regions. The Chinese Pharmacopoeia (2005 edition) listed *Cyrtomium fortumei* (J.) Smith as an official drug, which showed that the plant could be used as anticancer herbs [1]. In the past, people used the rhizomes and extracts from the plant as vermifuges [2]. The plant can be used to cure many diseases, such as influenza, acute and chronic pharyngitis, cancer, and migraine [3]. In addition, the herb was used as antiviral agents to cure severe acute respiratory syndrome, a life-threatening viral respiratory illness believed to be caused by a coronavirus [4]. Furthermore, phoroglucinols and flavonoids are known to display a wide array of pharmacological and biochemical actions, and have been isolated from many species of the Dryopteridaceae family [5]. However, so far the constituents with anticancer activity of the plant remain unclear. Thus it is necessary to identify the potent antitumor compounds from this plant.

We have recently investigated the chemical constituents of *Cyrtomium fortumei* (J.) Smith systematically, and tested the antitumor activities of the extracts and compounds. The current study was conducted to validate the medicinal use of *Cyrtomium fortumei* (J.) Smith. 23 compounds were isolated from the ethyl acetate and *n*-BuOH extracts, and their structures were elucidated to be protocate chaldehyde (**1**), woodwardinsauremethylester (**2**), pimpinellin (**3**), trans-2-coumaric acid (**4**), physcion (**5**), ursolic acid (**6**), sitost-4-en-3-one (**7**), betulin (**8**), 3^′^,4^′^,5-Trihydroxy-3,7-dimethoxyflavone (**9**), woodwardinic acid (**10**), sitosterol-3-O-β-D-glucopyranoside (**11**), sutchuenoside A (**12**), β-sitosterol (**13**), kaempferol-3,7-O-α-L-dirhamnoside (**14**), (-)-epicatchin (**15**), (+)-catechin hydrate (**16**), kaempferol (**17**), asiatic acid (**18**), 2β,3β,23-tihydroxy-12-oleanen-28-oic acid (**19**), crassirhizomoside A (**20**), kaempferol-3-O-(3-O-acetyl-α-L-rhamnopyranoside) (**21**), 2α,3α,24-trihydroxyurs-12-en-28-oic acid (**22**), and kaempferol-3-O-α-L-rhamnopyranoside-7-O-α-L-rhamopyranoside (**23**). All compounds were isolated from *Cyrtomium fortumei* (J.) Smith for the first time. The inhibitory activities of these compounds were investigated on tumor cells MGC-803, PC3, and A375 *in vitro* by MTT (thiazolyl blue tetrazolium bromide) assay, and the results indicated that some of the compounds showed good antitumor activities. It was found that pimpinellin (PPI) had most potent cytotoxic activities against the three cell lines, with the IC_50_ values of 14.4 ± 0.3 μM, 20.4 ± 0.5 μM, and 29.2 ± 0.6 μM, respectively. However, no report was found on apoptosis inducing activity of PPI. Subsequent staining and flow cytometry (FCM) analysis indicated that PPI could apoptosis in MGC-803 cells, with the highest apoptosis ratio of 27.44% at 72 h after treatment at 20 μM.

## Results and discussion

### Isolation and identification

Dried rhizomes of *Cyrtomium fortumei* (J.) Smith (10 kg) were cut into pieces and extracted with 80% EtOH (3 × 40 L) under reflux, 6 h for the first time, 3 h for the second time, and 1 h for the last time. The combined EtOH extracts were evaporated to dryness to yield a dried EtOH extract (424 g). The extract was suspended in water and then extracted with petroleum ether (10 L × 3 times), ethyl acetate (10 L × 3 times), and *n*-BuOH (10 L × 3 times), respectively. Different extracts solvent were then concentrated using evaporator under vacuum at 50°C to afford the petroleum ether extract (46 g), ethyl acetate extract (95 g), and *n*-BuOH extract (110 g). MTT assay was used to evaluate their antitumor activities. The results suggested that the antitumor agents were mainly contained in ethyl acetate and *n*-BuOH extracts. Further experiments were performed on ethyl acetate and *n*-BuOH extracts to separate antitumor compounds. The ethyl acetate and *n*-BuOH extracts were subjected to column chromatography (CC) to yield 16 fractions. An activity-directed isolation process was used to get compounds, and 23 compounds were obtained from these fractions.

The isolated compounds were identified *via* spectroscopic analyses, including ^1^H- and ^13^C-NMR spectroscopy. The results of the analyses were compared with the NMR and IR data reported in literatures to identify the chemical structures. All compounds were isolated from *Cyrtomium fortumei* (J.) Smith for the first time.

### Biological evaluation

**Figure 1:**
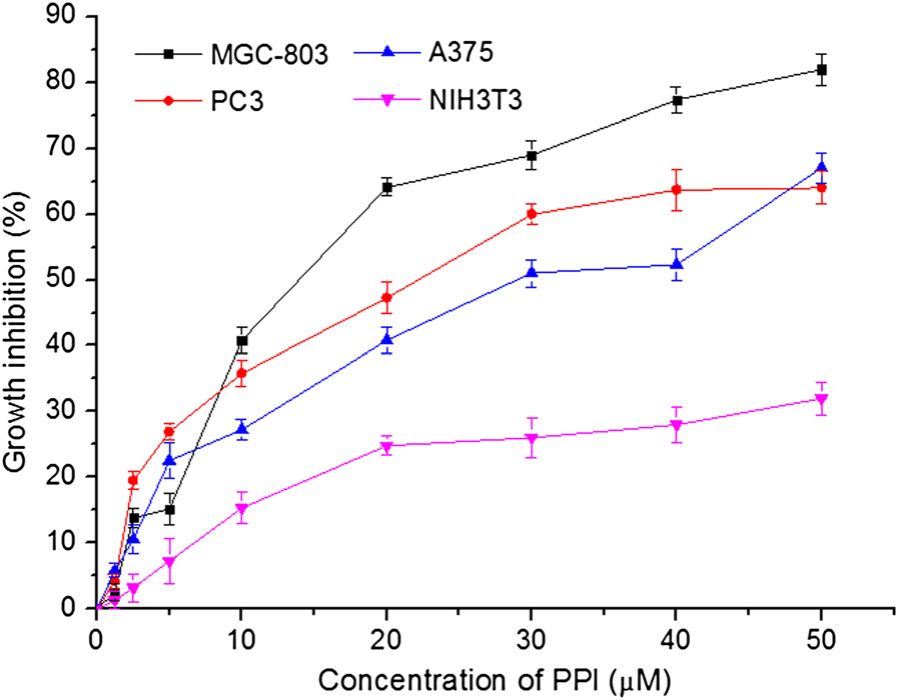
**Effect of PPI on proliferation of tumor cells. **After MGC-803, PC3, and A375 cells were treated with PPI for 72 h in the concentration varied from 1.25 to 50 μM, growth inhibition of those tumor cells was detected by MTT assay.

The potential effect of the extracts from *Cyrtomium fortumei* (J.) Smith was investigated on the viability of MGC-803, PC3, A375, and NIH3T3 cells using MTT assay at the concentration of 20 μM, with ADM (Adriamycin) being used as the positive control and DMSO being used as the negative control. The inhibitory percentage of cells was treated with 20 μM or 50 μg/mL of each compound or extract for 72 h (Table 1). The results showed that the ethyl acetate and *n*-BuOH extracts had great antitumor activity, and PPI had good activities against the three human cancer cell lines tested than other compounds. The inhibitory ratios of PPI at 72 h after treatment were 67.1% on MGC-803 cells, 57.2% on PC3 cells, 45.8% on A375 cells, and 24.8% on NIH3T3 cells. It could be seen that PPI had good antitumor activities and low cytotoxic effect on normal cell line NIH3T3. Further experiments found that proliferation of these three carcinoma cells were significantly inhibited by PPI in a concentration-dependent manner, as shown in Figure 1. The IC_50_ values of PPI against MGC-803, PC3, and A375 cells were determined to be 14.4 ± 0.3 μM, 20.4 ± 0.5 μM, and 29.2 ± 0.6 μM, respectively, which were lower than that on NIH3T3 cells (IC_50_ > 100 μM).

**Table 1:** **Growth inhibition effect of various constituents of *Cyrtomium fortumei *(J.) Smith on different cell lines**

**Test extracts and compounds**	**Inhibitory Rate for Different Cell Lines (%, mean ± SD)**^**a**^			
	**MGC-803**^**b**^	**PC3**^**c**^	**A375**^**d**^	**NIH3T3**^**e**^
Petroleum ether extract ^f^	56.7 ± 3.7	68.0 ± 1.9	60.5 ± 3.2	7.1 ± 3.8
Ethyl acetate extract ^f^	80.3 ± 2.8	89.6 ± 1.5	88.7 ± 3.3	45.6 ± 4.0
*n*-BuOH extract ^f^	87.2 ± 3.9	80.2 ± 2.4	85.2 ± 3.7	53.5 ± 8.6
ADM	93.2 ± 1.6	95.1 ± 2.6	95.2 ± 2.4	98.5 ± 1.7
Protocate chaldehyde (**1**)	17.8 ± 3.1	30.5 ± 2.4	25.7 ± 3.1	12.1 ± 3.7
Woodwardinsaure methylester (**2**)	47.9 ± 9.3	33.2 ± 2.8	41.4 ± 5.6	15.2 ± 2.6
PPI (**3**)	67.1 ± 5.3	57.2 ± 4.2	45.8 ± 7.8	24.8 ± 3.4
Physcion (**5**)	28.9 ± 2.2	20.7 ± 1.2	15.7 ± 5.9	4.5 ± 2.6
Ursolic acid (**6**)	52.6 ± 2.4	55.8 ± 3.1	43.2 ± 1.3	23.4 ± 3.3
Sitost-4-en-3-one (**7**)	35.8 ± 2.6	15.3 ± 5.8	26.4 ± 5.1	5.2 ± 2.7
Betulin (**8**)	45.1 ± 3.1	18.4 ± 3.1	33.9 ± 5.6	29.8 ± 2.5
3′,4′,5-Trihydroxy-3,7-dimethoxyflavone (**9**)	25.6 ± 3.9	20.3 ± 2.5	11.2 ± 6.2	10.0 ± 2.9
Woodwardinic acid (**10**)	25.1 ± 5.3	25.2 ± 1.3	38.6 ± 4.0	6.9 ± 2.0
Sutchuenoside A (**12**)	19.9 ± 9.8	12.3 ± 4.4	34.8 ± 5.3	8.8 ± 4.2
Kaempferol-3, 7-O-α-L-dirhamnoside (**14**)	21.5 ± 7.0	17.8 ± 5.5	36.7 ± 3.9	23.1 ± 3.2
(-)-Epicatchin (**15**)	45.5 ± 4.1	50.6 ± 1.6	45.3 ± 3.2	22.5 ± 3.8
(+)-Catechin hydrate (**16**)	52.1 ± 5.7	49.6 ± 2.3	46.8 ± 1.1	28.9 ± 4.3
Kampferol (**17**)	54.5 ± 2.4	44.3 ± 2.5	47.3 ± 1.9	12.2 ± 3.4
Crassirhizomoside A (**20**)	11.4 ± 2.3	20.8 ± 6.0	34.9 ± 2.7	12.5 ± 5.2
Kaempferol-3-O-(3-O-acetyl-α-L-rhamnopyranoside) (**21**)	22.6 ± 8.0	15.6 ± 6.1	17.8 ± 3.3	9.9 ± 6.1

Note: ^a ^Inhibitory percentage of cells treated with 20 μM of each compound for 72 h and SD = standard deviation; ^b^ Stomach cancer; ^c ^Prostate cancer; ^d ^Malignant melanoma; ^e ^Mouse fibroblasts; ^f ^Inhibitory percentage of cells treated with 200 μg/mL of each extract for 72 h.

To determine whether the growth inhibitory activity of PPI was related to the induction of apoptosis, the morphological character changes of MGC-803 cells were investigated using the AO/EB staining, Hoechst 33258 staining, and TUNEL assay.

The cell morphologic changes after PPI treatment were assessed by fluorescence microscopy after AO/EB staining [6]. AO is a vital dye that could penetrate the normal cell membrane [7], whereas EB will stain only those cells that have lost their membrane integrity [8]. The stained cells revealed four different types under a fluorescence microscope: the chromatin of living cells was green with normal structure; the chromatin of non-apoptotic dead cells was red with normal structure; yellow coloration of early apoptotic cells with morphous in the form of pycnosis; and orange coloration of late apoptotic cells with morphous in the form of pycnosis [9-13]. With hydroxycamptothecine (HCPT) as positive control, the cytotoxicity of PPI at a concentration of 20 μM against MGC-803 cells for 24 h was detected *via* AO/EB staining.

**Figure 2:**
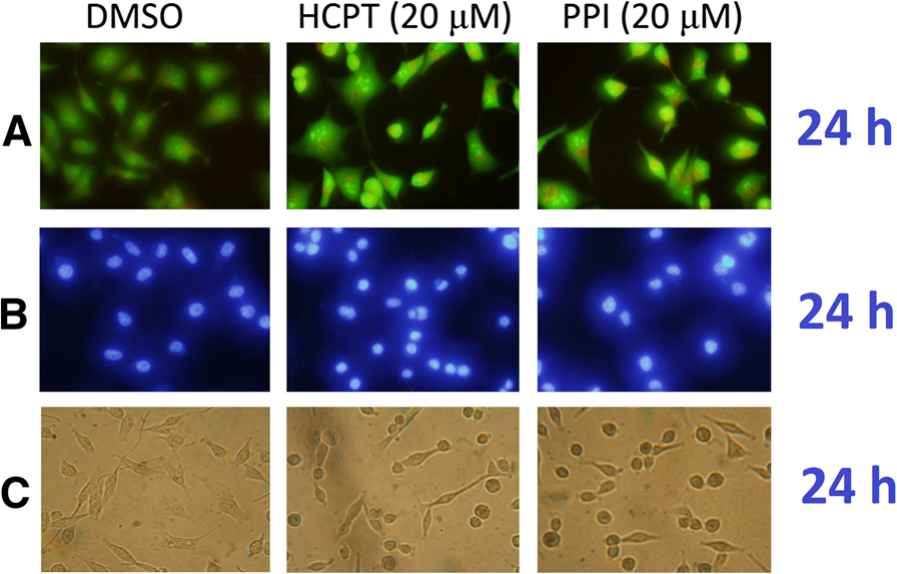
**Apoptosis induction studies of PPI. **(**A**) AO\EB staining. (**B**) Hoechst 33258 staining. (**C**) TUNEL assay.

As can be seen in Figure 2A, green live MGC-803 cells with normal morphology were seen in the negative control group. Green yellow or orange dots were detected in the HCPT after 24 h. After cells were treated with PPI for 24 h, the nuclei stained as yellow green or orange, and the morphology showed pycnosis, membrane blebbing and cell budding. These phenomena indicate PPI could induce apoptosis without any significant cytotoxicity.

Live cells with uniformly light blue nuclei were treated with Hoechst 33258 [14-17] and observed under a fluorescence microscope. However, apoptotic cells had bright blue nuclei because of karyopyknosis and chromatin condensation; the nuclei of dead cells could not be stained [18-22]. With hydroxycamptothecine (HCPT) as positive control, MGC-803 cells treated with PPI at 20 μM for 24 h were stained with Hoechst 33258.

As can be seen in Figure 2B, the cells of the negative group (DMSO) were normal blue. However, the cells of HCPT treatment appeared to be compact and condensed. After treatment with PPI for 24 h, most of cell nuclei appeared to be highly condensed and crescent-shaped, indicating that PPI induced apoptosis against MGC-803 cell lines. These results were consistent with AO/EB double staining.

In addition, TUNEL, one of the popular methods, identified apoptotic cells *in situ via* the detection of DNA fragmentation [23,24]. Under a biological microscope, the cells were observed that where brown precipitate was the result of positive apoptosis [25-27]. MGC-803 cells were treated PPI and HCPT 20 μM at with for 24 h.

As can be seen in Figure 2C, the cells of the negative group (DMSO) did not appear as brown precipitates, whereas both PPI and HCPT appeared as brown precipitates. Therefore, it can be further concluded that PPI induced apoptosis against MGC-803 cells. The results were identical with the previous experiment.

**Figure 3:**
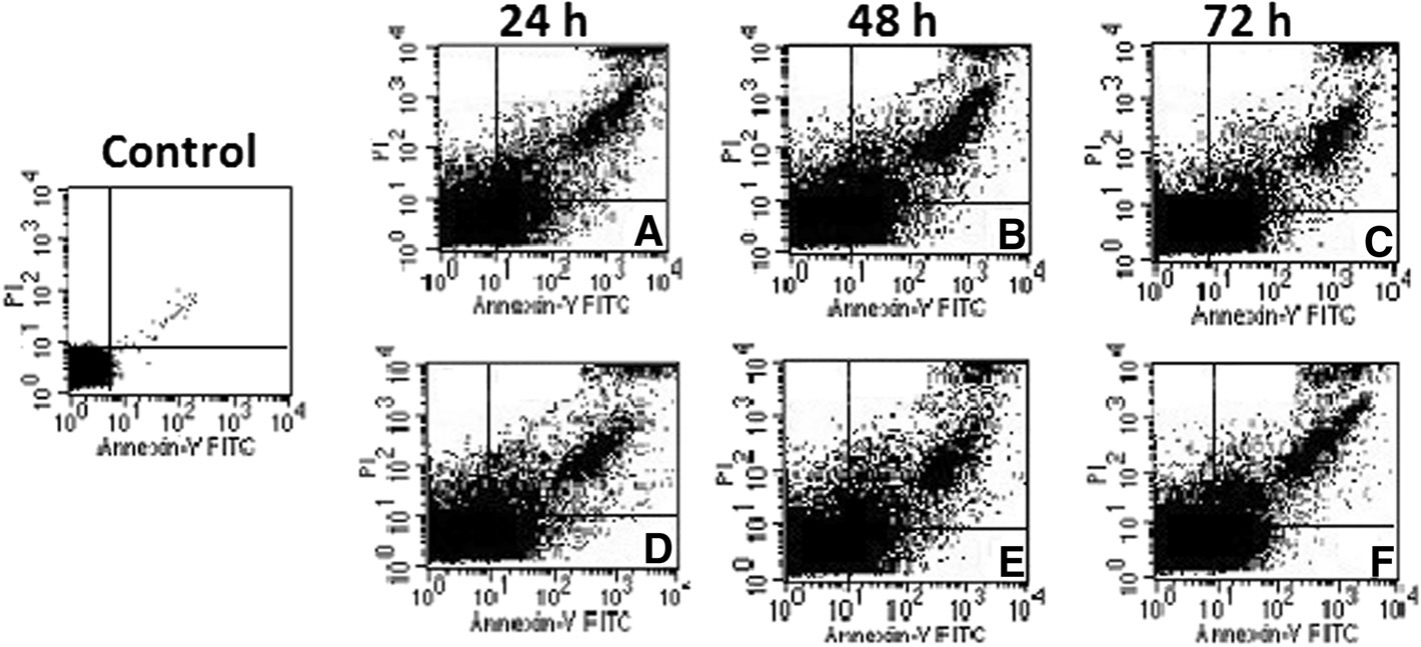
**FCM tested apoptosis detection assay on MGC-803 cells. **The appearance of apoptosis cells was detected by flow cytometry using Annexin V/PI staining. In the figure, **A**, **B**, and **C**: treat with HCPT (20 μM) for 24, 48, and 72 h; **D**, **E**, and **F**: treat with PPI (20 μM) for 24, 48, and 72 h.

**Figure 4:**
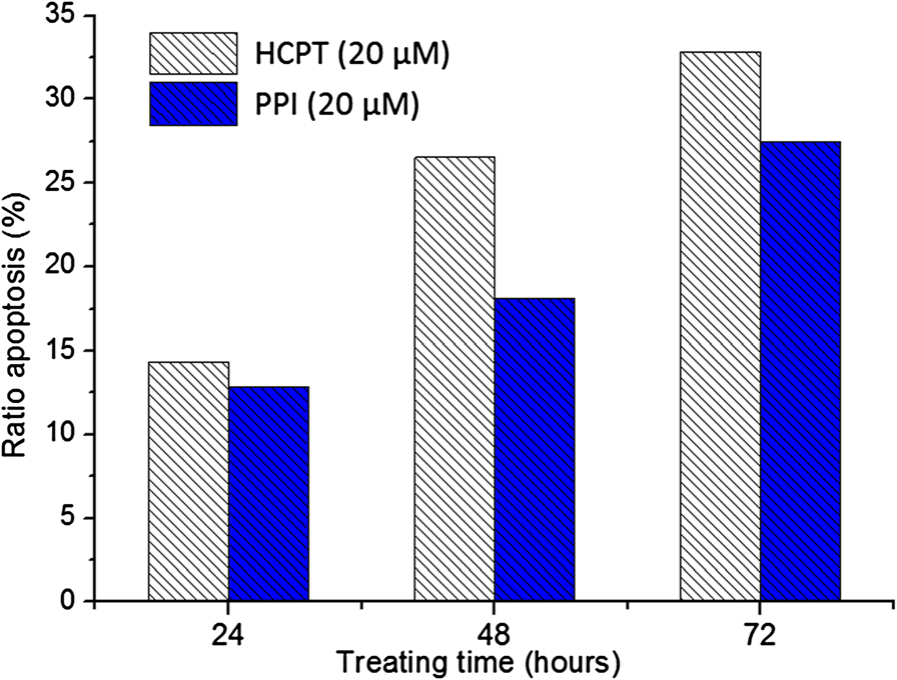
**Effects of concentration of PPI on apoptosis. **These cells were treated with HCPT and PPI for 24, 48, and 72 h.

The apoptosis ratios induced by PPI in tumor cells was quantitatively assessed by FCM [28]. In early apoptotic cells, phosphatidylserine (PS) which distributed inside the lipid bilayer in the normal cells was transferred from the inside of the cell membrane to the outside. Annexin V, a Ca^2+^ dependent phospholipid-binding protein with a high affinity for PS, was used to detect early apoptotic cells. PI (Propidine Iodide) was a red fluorescent dye and stained cells that had lost membrane integrity. So, the different periods of apoptotic cells could be distinguished when Annexin V matched with PI: necrotic cells (the upper left quadrant, Annexin^-^/PI^+^), late apoptotic cells (the upper right quadrant, Annexin^+^/PI^+^), intact cells (the lower left quadrant, Annexin^-^/PI^-^) and early apoptotic cells (the lower right quadrant, Annexin^+^/PI^-^) [29,30]. As shown in Figure 3, with HCPT as positive control, PPI could induce apoptosis of MGC-803 cells, with the highest apoptosis ratio of 27.44% at 72 h after treatment at 20 μM. Furthermore, as shown in Figure 4 the apoptosis of MGC-803 which treated with PPI increased gradually in a time-dependent manner.

## Conclusions

In this study, an activity-directed fractionation and purification process were used to isolate antitumor compounds from the rhizomes of *Cyrtomium fortumei* (J.) Smith. The ethyl acetate and *n*-BuOH extracts had good antitumor activities, consequently leading to the isolation of 23 compounds identified as protocate chaldehyde (**1**), woodwardinsauremethylester (**2**), pimpinellin (**3**), trans-2-coumaric acid (**4**), physcion (**5**), ursolic acid (**6**), sitost-4-en-3-one (**7**), betulin (**8**), 3^′^,4^′^,5-trihydroxy-3,7-dimethoxyflavone (**9**), woodwardinic acid (**10**), sitosterol-3-O-β-D-glucopyranoside(**11**), sutchuenoside A (**12**), β-sitosterol (**13**), kaempferol-3,7-O-α-L-dirhamnoside (**14**), (-)-epicatchin (**15**), (+)-catechin hydrate (**16**), kaempferol (**17**), asiatic acid (**18**), 2β,3β,23-tihydroxy-12-oleanen-28-oic acid (**19**), crassirhizomoside A (**20**), kaempferol-3-O-(3-O-acetyl-α-L-rhamnopyranoside) (**21**), 2α,3α,24-trihydroxyurs-12-en-28-oic acid (**22**), and kaempferol-3-O-α-L-rhamnopyranoside-7-O-α-L-rhamopyranoside (**23**). All compounds were isolated from *Cyrtomium fortumei* (J.) Smith for the first time. The inhibitory activities of these compounds were investigated on tumor cells MGC-803, PC3, and A375 *in vitro* by MTT assay, and the results showed that some of the compounds exhibited moderate antiproliferative activities. PPI showed potent activities on MGC-803, PC3, and A375 cell lines, with the IC_50_ values of 14.4 ± 0.3 μM, 20.4 ± 0.5 μM, and 29.2 ± 0.6 μM, respectively. The apoptosis inducing activities of PPI on MGC-803 cell lines were investigated through AO/EB staining, Hoechst 33258 staining, TUNEL and FCM assay. The results demonstrated that PPI could induce cell apoptosis of MGC-803 cells. Further studies of the specific mechanisms of the compound on human malignant tumors are currently underway.

## Experimental

### General procedures and reagents

The melting points of the products were determined using an XT-4 binocular microscope (Beijing Tech Instrument Co. Ltd., Beijing, China). Infrared spectra were recorded on a Bruker VECTOR22 spectrometer in KBr disks. ^1^H-NMR and ^13^C-NMR were recorded using a JEOL-ECX500 spectrometer at 22°C, with tetramethylsilane as the internal standard and CDCl_3_, DMSO-*d*_6_, CD_3_COCD_3_, or CD_3_OD as the solvent. Column chromatography was performed using silica gel (200–300 meshes) (Qingdao Marine Chemistry Co., Qingdao, China) and silica gel H (Qingdao Marine Chemistry Co., Qingdao, China), Sephadex LH-20 (GE Healthcare Bio-Sciences AB, Uppsala, Sweden), HP-20 (Mitsubishi Chemical Corp., Toukyu Met, Japan), YMC RP-18 (YMC Corp., Kyoto, Japan) and MCI-gel CHP 20P (Mitsubishi Chemical Corp., Toukyu Met, Japan). All other chemicals were of analytical reagent grade and used without further purification.

### Plant materials

Fresh samples of *Cyrtomium fortumei* were collected from Longli, Guizhou Province in China, in August 2011. Prof. Qingde Long, Department of Medicine, Guiyang Medical University, identified the plant material. A voucher specimen was deposited at Guiyang Medical University, Guiyang, China.

### Extraction and isolation

Dried rhizomes of *Cyrtomium fortumei* (J.) Smith (10 kg) were cut into pieces and extracted with 80% EtOH (3 × 40 L) under reflux, 6 h for the first time, 3 h for the second time and 1 h for the last time. The combined EtOH extracts were evaporated to dryness to yield a dried EtOH extract (424 g). The extract was suspended in water and then extracted with petroleum ether (10 L × 3 times), ethyl acetate (10 L × 3 times) and *n*-BuOH (10 L × 3 times) respectively. The organic ethyl acetate extract (95 g) was subjected to CC on silica gel (200-300 mesh) eluted with a gradient of petroleum ether-EtOAc (20/1, 10/1, 5/1, 2/1 and 1/1, *v/v*), and finally a mixture of chloroform and methanol (10/1, 5/1 and 1/1, *v/v*, respectively) to get 10 fractions, namely, fraction 1-10. The fractions were monitored by TLC. Fraction 3 (10 g) was applied to a silica gel (200-300 mesh) column eluted with petroleum ether-EtOAc (10:1 to 1:1) to yield Protocate chaldehyde (**1**, 30 mg), Woodwardinsauremethylester (**2**, 13 mg) and yield 7 fractions, namely, Fraction 1^′^-7^′^. Fractions 2^′^ and 5^′^ were purified using PTLC to yield Pimpinellin (**3**, 25 mg), Trans-2-coumaric acid (**4**, 13 mg), and Physcion (**5**, 18 mg). Fractions 6^′^ and 7^′^ was separated using silica gel (300-400 mesh) eluted with petroleum ether-EtOAc (10:1) to yield Ursolic acid (**6**, 23 mg), Sitost-4-en-3-one (**7**, 14 mg), and Betulin (**8**, 18 mg). Fraction 5 (8 g) was applied to a silica gel H column eluted with CHCl_3_-MeOH (10:1 to 5:1) to yield a yellow powder (3 g), and purified using a Sephadex LH-20 column eluted with MeOH to yield 3^′^,4^′^,5-Trihydroxy-3,7-dimethoxyflavone (**9**, 15 mg). The yellow powder was purified using silica gel repeatedly, to yield Woodwardinic acid (**10**, 32 mg), Sitosterol-3-O-β-D-glucopyranoside (**11**, 43 mg), and Sutchuenoside A (**12**, 16 mg). Fraction 6 (13 g) was applied to a silica gel H column eluted with CHCl_3_-MeOH (8:1 to 2:1) to yield 2 fractions, namely fraction A and B. Fraction A was purified using a Sephadex LH-20 column eluted with CHCl_3_-MeOH (1:1) to yield β-sitosterol (**13**, 24 mg), and kaempferol-3,7-O-α-L-dirhamnoside (**14**, 25 mg). Fraction B was recrystallized with acetone to give compound (-)-Epicatchin (**15**, 13 mg).

The *n*-BuOH extract (110 g) was suspended in H_2_O, adsorbed on an MCI-gel column (500 g), and eluted using mixtures of H_2_O and MeOH starting with 20% MeOH (*v/v*) to 100% MeOH in 10% increments (2.5 L each fraction) to afford a total of 6 fractions, namely fraction 11-16. The fractions were also monitored by RP-TLC. Fraction 12 (6 g) was decolorized and separated on MCI-gel eluted with 50-70% MeOH-H_2_O to yield (+)-Catechin hydrate (**16**, 30 mg) and Kaempferol (**17**, 12 mg). Fraction 13 (12 g) was further chromatographed on a YMC RP-18 column (50 μm) and eluted using a MeOH-H_2_O gradient solvent system (50:50 to 90:10) to yield Asiatic acid (**18**, 14 mg), 2β,3β,23-tihydroxy-12-oleanen-28-oic acid (**19**, 16 mg), and a yellow powder (2 g). The yellow powder was further purified over a Sephadex LH-20 column and eluted with CHCl_3_-MeOH (1:1) to yield Crassirhizomoside A (**20**, 22 mg) and subfraction (A1-A4). Fraction A2 was separated using silica gel (300-400 mesh) and purified through Sephadex LH-20 to yield Kaempferol-3-O-(3-O-acetyl-α-L-rhamnopyranoside) (**21**, 14 mg). Fraction A3 was purified with silica gel (300-400 mesh) to yield 2α,3α,24-trihydroxyurs-12-en-28-oic acid (**22**, 16 mg). Fraction 14 (11 g) were separated using HP-20 resin eluted with 60%-90% MeOH-H_2_O and purified using a Sephadex LH-20 column eluted with MeOH to yield Kaempferol-3-O-α-L-rhamnopyranoside-7-O-α-L-rhamopyranoside (**23**, 23 mg).

All spots on TLC were visualized by heating silica gel plates sprayed with 10% Phosphomolybdic acid hydrate in EtOH and 1% FeCl_3_ in EtOH.

### Spectroscopic data

Protocate chaldehyde (**1**). Yellow powder, mp 151-153°C. ^1^H-NMR (CD_3_OD, 500 MHz) δ: 9.63 (1H, s, CHO), 7.23 (2H, m, H-6, -2), 6.86 (1H, d, *J* = 5 Hz, H-5). ^13^C-NMR (CD_3_OD, 125 MHz) δ: 192.1 (-CHO), 152.4 (C-4), 145.7 (C-3), 129.3 (C-1), 125.49 (C-6), 115.0 (C-5), 114.3 (C-2). The above data were identical to the literature data [31].

Woodwardinsauremethylester (**2**). White powder, mp 201-203°C. IR (KBr, cm^−1^) *ν*_max_: 3480, 1738, 1630, 890. ^1^H-NMR (CDCl_3_, 500 MHz) δ: 4.75 (2H, s, H-30), 3.67 (3H, s, COOCH_3_), 1.63 (3H, s, Me), 1.17 (3H, s, Me), 0.99 (3H, s, Me), 0.85 (3H, s, Me), 0.83 (3H, s, Me), 0.61 (3H, s, Me). ^13^C-NMR (CDCl_3_, 125 MHz) δ: 181.2(COOCH_3_), 148.8 (C-30), 109.8 (C-22), 66.4 (C-3), 57.3 (COOCH_3_), 19.7 (C-29), 19.5 (C-26), 17.8 (C-27), 16.6 (C-28), 16.3 (C-25), 16.0 (C-24), The above data were identical to the literature data [32].

Pimpinellin (**3**). Pale yellow powder, mp 118-120°C. IR (KBr, cm^−1^) *ν*_max_: 1724, 1613, 1576, 1060. ^1^H-NMR (CDCl_3_, 500 MHz) δ: 8.07 (1H, d, *J* = 10 Hz, H-4), 7.64 (1H, d, *J* = 1.5 Hz, H-12), 7.05 (1H, d, *J* = 3 Hz,H-11), 6.34 (1H, d, *J* = 10 Hz,H-3), 4.12 (3H, s, OCH_3_), 4.01 (3H, s, OCH_3_). ^13^C-NMR (CD_3_OD, 125 MHz) δ: 160.9 (C-2), 149.8 (C-7), 145.4 (C-12), 144.4 (C-9), 143.2 (C-5), 140.0 (C-4), 135.2 (C-6), 114.2 (C-3), 113.8 (C-8), 109.5 (C-10), 104.3 (C-11), 62.4 (OCH_3_), 61.3 (OCH_3_). The above data were identical to the literature data [33].

Trans-2-coumaric acid (**4**). White powder, mp 189-191°C. ^1^H-NMR (CD_3_OD, 500 MHz) δ: 7.99 (1H, d, *J* = 1.5 Hz, H-1^′^), 7.19 (1H, m, H-4), 6.94 (1H, m, H-4), 6.86 (1H, m, H-3), 6.57 (1H, d, *J* = 2 Hz, H-2^′^). ^13^C-NMR (CD_3_OD, 125 MHz) δ: 168.0 (C = O), 156.6 (C-2), 140.5 (C-1^′^), 131.5 (C-4), 128.9 (C-6), 121.5 (C-1), 120.0 (C-5), 117.9 (C-3), 116.2 (C-2^′^). The above data were identical to the literature data [34].

Ursolic acid (**6**). White powder, mp 257–259°C. IR (KBr, cm^−1^) *ν*_max_: 3444, 2928, 2868, 1683, 1458, 1028. ^1^H-NMR (CD_3_OD, 500 MHz) δ: 0.78 (CH_3_, d, *J* = 10 Hz, H-29), 0.84 (CH_3_, d, *J* = 10 Hz, H-30), 0.90 (CH_3_, s, H-23), 0.92 (CH_3_, s, H-26), 0.97 (CH_3_, s, H-25), 1.09 (CH_3_, s, H-27), 3.20 (1H, d, *J* = 5 Hz, H-3), 5.2 (1H, t, *J* = 1.5 Hz, H-12). ^13^C-NMR (CD_3_OD, 125 MHz) δ: 180.1 (C-28), 138.3 (C-13), 125.6 (C-12), 78.1 (C-3), 55.4 (C-5), 53.1 (C-18), 41.9 (C-14), 39.5 (C-8), 39.1 (C-19), 38.9 (C-1), 38.7 (C-4), 38.5 (C-20), 36.8 (C-10), 36.7 (C-22), 33.0 (C-7), 30.4 (C-21), 27.9 (C-23), 27.4 (C-15), 26.6 (C-2), 24.2 (C-16), 23.0 (C-11), 22.7 (C-27), 21.2 (C-30), 18.3 (C-6), 16.5 (C-26), 16.2 (C-29), 14.7 (C-25), 14.6 (C-24). The above data were identical to the literature data [35].

Sitost-4-en-3-one (**7**). White crystalline, mp 90-92°C. IR (KBr, cm^−1^) *ν*_max_: 2940, 1688, 1390, 867. ^1^H-NMR (CDCl_3_, 500 MHz) δ: 5.58 (1H, d, *J* = 1.5 Hz, H-4), 1.07 (3H, s, H-19), 0.88 (3H, m, H-29), 0.71 (3H, s, H-18). ^13^C-NMR (CD_3_OD, 125 MHz) δ: 199.6 (C-3), 171.74 (C-5), 123.68 (C-4), 55.9 (C-17), 55.8 (C-14), 53.7 (C-9), 45.8 (C-24), 42.3 (C-13), 39.5 (C-12), 38.6 (C-10), 36.1 (C-20), 35.6 (C-8), 35.5 (C-1), 33.9 (C-22), 33.9 (C-2), 32.9 (C-6), 32.0 (C-7), 29.1 (C-25), 28.1 (C-16), 26.0 (C-23), 24.1 (C-15), 23.0 (C-28), 21.0 (C-11), 19.8 (C-26), 19.0 (C-27), 18.7 (C-21), 17.3 (C-19), 11.9 (C-29), 11.9 (C-18). The above data were identical to the literature data [36].

Betulin (**8**). White powder, mp 234–236°C. IR (KBr, cm^−1^) *ν*_max_: 3498, 2941, 1456, 1033. ^1^H-NMR (CDCl_3_, 500 MHz) δ: 3.79 (1H, d, *J* = 10 Hz, H-3), 4.67 (1H, s, H-29), 4.56 (1H, s, H-29), 3.77 (1H, d, *J* = 5 Hz, H-28), 3.31 (1H, d, *J* = 10 Hz, H-28), 3.18 (1H, d, *J* = 5.5 Hz, H-3), 1.66 (3H, s, H-30). ^13^C-NMR (CDCl_3_, 125 MHz) δ: 150.5 (C-20), 109.7 (C-29), 79.0 (C-3), 60.6 (C-28), 55.3 (C-5), 50.4 (C-9), 48.8 (C-18), 47.8 (C-17, 19), 42.7 (C-14), 40.9 (C-8), 38.9 (C-4), 38.7 (C-1), 37.4 (C-13), 37.2 (C-10), 34.2 (C-7), 34.0 (C-22), 29.8 (C-21), 29.2 (C-16), 28.0 (C-23), 27.4 (C-2), 27.1 (C-15), 25.3 (C-12), 20.8 (C-11), 19.1 (C-30), 18.4 (C-6), 16.1 (C-25), 16.0 (C-26), 15.4 (C-24), 14.8 (C-27). The above data were identical to the literature data [37].

3^′^,4^′^,5-Trihydroxy-3,7-dimethoxyflavone (**9**). Yellow powder, mp 233-235°C. IR (KBr, cm^-1^) *ν*_max_ 3455, 1642, 1531. ^1^H-NMR (CD_3_OD, 500 MHz) δ: 7.47 (1H, d, *J* = 3 Hz, H-2^′^), 6.85 (1H, d, *J* = 10 Hz, H-5^′^), 6.45 (1H, d, *J* = 2.5 Hz, H-8), 6.35 (1H, d, *J* = 3 Hz, H-6), 3.86 (3H, s, OCH_3_) 3.73 (3H, s, OCH_3_). ^13^C-NMR (CD_3_OD, 125 MHz) δ: 174.8 (C-4), 162.4 (C-7), 161.1 (C-9), 158.8 (C-5), 154.8 (C-2), 148.1 (C-4^′^), 145.0 (C-3^′^), 140.0 (C-3), 121.7 (C-1^′^), 120.6 (C-6^′^), 115.0 (C-2^′^), 114.9 (C-5^′^), 107.3 (C-10), 95.7 (C-6), 94.6 (C-8), 58.8 (OCH_3_), 55.1 (OCH_3_). The above data were identical to the literature data [38].

Woodwardinic acid (**10**). White powder, mp 242-244°C. IR (KBr, cm^−1^) *ν*_max_: 3490, 1709, 1635, 886. ^1^H-NMR (DMSO-*d*_*6*_, 500 MHz) δ: 4.75 (2H, s, H-30), 1.69 (3H, s, Me), 1.24 (3H, s, Me), 1.10 (3H, s, Me), 0.95 (3H, s, Me), 0.92 (3H, s, Me), 0.69 (3H, s, Me). ^13^C-NMR (DMSO-*d*_*6*_, 125 MHz) δ: 180.3 (C-23), 148.3 (C-30), 110.3 (C-22), 65.7 (C-3), 21.2 (C-29), 18.9 (C-27), 18.4 (C-26), 16.3 (C-28), 16.0 (C-24), 16.0 (C-25). The above data were identical to the literature data [39].

Sutchuenoside A (**12**). Pale yellow amorphous powder, mp 211-213°C. IR (KBr, cm^−1^) *ν*_max_: 3455, 1701, 1623. ^1^H-NMR (CD_3_OD, 500 MHz) δ: 7.74 (2H, d, *J* = 9 Hz, H-2^′^, -6^′^), 6.94 (2H, d, *J* = 9 Hz, H-3^′^, -5^′^), 6.71 (1H, d, *J* = 1 Hz, H-8), 6.45 (1H, d, *J* = 1 Hz, H-6), 5.57 (1H, br s, H-1″), 5.50 (1H, s, 2‴-OH), 4.52 (1H, s, 3‴-OH), 4.19 (1H, br s, H-2″), 3.92 (1H, br s, H-2‴), 3.82 (1H, m, H-3″), 3.58 (1H, m, H-3‴), 3.45 (1H, m, H-5‴), 3.21-3.25 (2H, m, H-4‴, -5″), 2.08 (3H, s, Ac), 1.25 (3H, d, *J* = 6.5 Hz, Me-6‴), 0.77 (3H, d, *J* = 5 Hz, Me-6″). ^13^C-NMR (CD_3_OD, 125 MHz) δ: 178.3 (C-4), 171.0 (COCH_3_), 162.2 (C-7), 161.6 (C-5), 160.5 (C-4^′^), 158.6 (C-2), 156.8 (C-9), 134.5 (C-3), 130.7 (C-3^′^,-5^′^), 120.9 (C-1^′^), 115.2 (C-2^′^,-6^′^), 106.2 (C-10), 101.2 (C-1^′^), 99.3 (C-6), 98.5 (C-1‴), 94.3 (C-8), 73.5 (C-4″), 72.2 (C-4‴), 70.7 (C-3‴), 70.3 (C-5‴), 70.3 (C-2″), 69.9 (C-2‴), 68.6 (C-5″), 68.3 (C-3″), 19.6 (COCH_3_), 17.0 (C-6″), 16.2 (C-6″). The above data were identical to the literature data [40].

Kaempferol-3,7-O-α-L-dirhamnoside (**14**). Light yellow crystals, mp 175-177°C. IR (KBr, cm^−1^) *ν*_max_: 3347, 2912, 1613, 876. ^1^H-NMR (CD_3_OD, 500 MHz) δ: 7.78 (2H , d, *J* = 8.5 Hz), 6.92 (2H , d, *J* = 8.5 Hz), 6.70(1H, d, *J* = 2 Hz), 6.44 (1H, d, *J* = 2.5 Hz), 5.54 (1H, br s, H-1″), 5.38 (1H, br s, 2‴-OH), 1.24 (3H, d, *J* = 6 Hz, Me-6‴), 0.91(3H, d, *J* = 5.5 Hz, Me-6‴). ^13^C-NMR (CD_3_OD, 125 MHz) δ: 178.4 (C-4), 162.2 (C-7), 160.4 (C-4^′^), 158.4 (C-2), 156.7 (C-9), 135.1 (C-3), 162.2 (C-5), 99.2 (C-6), 94.2 (C-8), 121.0 (C-1^′^), 115.2 (C-2^′^,-6^′^), 130.7 (C-3^′^,-5^′^), 106.2 (C-10), 102.1 (C-1″), 98.5 (C-1‴), 72.2 (C-4″), 71.8 (C-4‴), 70.7 (C-2″), 70.7 (C-5‴), 70.7 (C-3‴), 70.5 (C-2‴), 70.3 (C-5″), 69.9 (C-3″), 16.7 (C-6‴), 16.3 (C-6″). The above data were identical to the literature data [41].

(-)-Epicatchin (**15**). White powder, mp 222-224°C. IR (KBr, cm^-1^) *ν*_max_ 3440, 1622, 1461, 1289; ^1^H NMR (CD_3_OD, 500 MHz) δ: 6.79 (1H, d, *J* = 5 Hz, H-6^′^), 6.75 (1H, d,  = 15 Hz, H-2^′^), 6.70 (1H, d, *J* = 1.5 Hz, H-5), 5.93 (1H, d, *J* = 3 Hz, H-8), 5.84 (1H, d, *J* = 1.5 Hz, H-6), 4.80 (1H, s, H-2), 2.85 (1H, dd, *J* = 10 Hz, 12.5 Hz, H-3), 2.48 (1H, dd, *J* = 12 Hz, 10 Hz, H-4); ^13^C NMR (CD_3_OD, 125 MHz) δ: 156.8 (C-9), 156.2 (C-7), 155.5 (C-5), 144.8 (C-3^′^), 144.9 (C-4^′^), 130.9 (C-1^′^), 118.0 (C-6^′^), 114.7 (C-2^′^), 113.9 (C-5^′^), 98.7 (C-10), 95.0 (C-6), 94.5 (C-8), 78.5 (C-2), 66.1 (C-3), 27.9 (C-4). The above data were identical to the literature data [42].

(+)-Catechin hydrate (**16**). White powder, mp 134-136°C. IR (KBr, cm^-1^) *ν*_max_ 3350, 1635, 1511; ^1^H-NMR (CD_3_OD, 500 MHz) δ: 6.81 (1H, d, *J* = 1 Hz, H-2^′^), 6.72 (1H, d, *J* = 8 Hz, H-6^′^), 6.70 (1H, d, *J* = 1.5 Hz, H-5), 5.90 (1H, d, *J* = 3 Hz, H-8), 5.82 (1H, d, *J* = 2 Hz, H-6), 4.54 (1H, d, *J* = 7 Hz, H-2), 2.83 (1H, dd, *J* = 5, 15 Hz, H-3), 2.47 (1H, dd, *J* = 14, 10 Hz, H-4).^13^C-NMR (CD_3_OD, 125 MHz) δ: 156.5 (C-7), 156.2 (C-9), 155.6 (C-5), 144.9 (C-4^′^), 144.9 (C-3^′^), 130.8 (C-1^′^), 118.7 (C-6^′^), 114.7 (C-5^′^), 113.9 (C-2^′^), 99.5 (C-10), 94.9 (C-6), 94.1 (C-8), 81.5 (C-2), 67.4 (C-3), 27.2 (C-4). The above data were identical to the literature data [43].

Kampferol (**17**). Yellow powder, mp 279-281°C. IR (KBr, cm^-1^) *ν*_max_ 3409, 1649, 1511; ^1^H-NMR (CD_3_OD, 500 MHz) δ: 12.48 (1H, s, C-5-OH), 7.98 (2H, d, *J* = 1.5 Hz, H-2^′^, H-6^′^), 6.88 (2H, d, *J* = 4 Hz, H-3^′^, H-5^′^), 6.35 (1H, d, *J* = 1 Hz, H-8), 6.11 (1H, d, *J* = 1.5 Hz, H-6). ^13^C-NMR (CD_3_OD, 125 MHz) δ: 175.8 (C-4), 164.1 (C-7), 161.2 (C-9), 159.2 (C-4^′^), 156.9 (C-5), 146.2 (C-2), 135.8 (C-6^′^), 129.6 (C-2^′^), 122.5 (C-1^′^), 115.5 (C-5^′^), 115.4 (C-3^′^), 103.3 (C-10), 98.3 (C-6), 93.7(C-8). The above data were identical to the literature data [44].

Asiatic acid (**18**). White powder, mp 233-235°C. IR (KBr, cm^−1^) *ν*_max_: 3428, 3380, 1688, 1375, 920. ^1^H-NMR (CD_3_OD, 500 MHz) δ: 1.06 (3H, s, H-25), 0.99 (9H, s, H-24, 26, 27), 0.90 (3H, d, *J* = 8 Hz, H-29), 0.84 (3H, d, *J* = 1.5 Hz, H-30). ^13^C-NMR (CD_3_OD, 125 MHz) δ: 179.7 (C-28), 139.1 (C-13), 125.4 (C-12), 77.9 (C-3), 68.7 (C-2), 66.3 (C-23), 53.4 (C-18), 47.9 (C-17), 47.9 (C-5), 47.7 (C-9), 47.7 (C-1), 43.4 (C-14), 42.3 (C-4), 39.9 (C-8), 39.8 (C-20), 39.3 (C-19), 39.2 (C-10), 37.2 (C-22), 33.0 (C-7), 30.9 (C-21), 28.4 (C-15), 24.7 (C-16), 23.7 (C-11), 23.5 (C-27), 21.2 (C-30), 18.3 (C-6), 17.3 (C-29), 17.3 (C-26), 17.3 (C-25), 14.3 (C-24). The above data were identical to the literature data [45].

2β,3β,23-tihydroxy-12-oleanen-28-oic acid (**19**). White powder, mp 212-215°C. IR (KBr, cm^−1^) *ν*_max_: 3410-3385, 1688, 1355. ^1^H-NMR (CD_3_OD, 500 MHz) δ: 5.38 (H, br s, H-12), 3.72 (1H, br s, H-3), 3.21 (1H, dd, *J* = 4, 10.5 Hz, H-18), 1.25, 1.22, 1.11, 1.05, 0.96 (15H, s, H-25, 26, 27, 29, 30). ^13^C-NMR (CD_3_OD, 125 MHz) δ: 180.0 (C-28), 144.6 (C-13), 122.3 (C-12), 74.0 (C-3), 66.0 (C-24), 65.0 (C-2), 53.3 (C-18), 49.2 (C-5), 47.8 (C-9), 46.2 (C-17), 45.0 (C-4), 41.7 (C-1), 41.4 (C-14), 39.2 (C-10), 39.2 (C-8), 39.8 (C-19), 34.0 (C-22), 33.8 (C-21), 33.5 (C-7), 33.0 (C-29), 30.9 (C-20), 28.4 (C-15), 25.9 (C-27), 25.0 (C-16), 23.9 (C-23), 23.9 (C-11), 23.7 (C-30), 17.3 (C-6), 17.0 (C-25), 16.9 (C-26). The above data were identical to the literature data [46].

Crassirhizomoside A (**20**). Pale yellow amorphous powder, mp 192-194°C. IR (KBr, cm^−1^) *ν*_max_: 3428, 1745, 1655, 1610. ^1^H-NMR (CD_3_OD, 500 MHz) δ: 7.73 (2H, d, *J* = 10 Hz, H-2^′^, 6^′^), 6.93 (2H, d, *J* = 10 Hz, H-3^′^, 5^′^), 6.71 (1H, br s, H-8), 6.44 (1H, br s, H-6), 2.09 (3H, s, COCH_3_-4″), 2.02 (3H, s, COCH_3_-2″), 1.24 (3H, d, *J* = 2.5 Hz, H-6‴), 0.80 (3H, d, *J* = 3.5 Hz, H-6″). ^13^C-NMR (CD_3_OD, 125 MHz) δ: 178.0 (C-4), 170.9 (4″-COCH_3_), 170.8 (2″-COCH_3_), 162.2 (C-5), 162.2 (C-4^′^), 161.6 (C-7), 160.6(C-2), 156.7 (C-9), 133.9 (C-3), 130.6 (C-6^′^), 130.6 (C-2^′^), 120.7 (C-1^′^), 115.3 (C-5^′^), 115.3 (C-3^′^), 106.1 (C-10), 100.5 (C-6), 99.3 (C-1″), 98.5 (C-1‴), 94.3 (C-8), 73.3 (C-4″), 73.3 (C-4‴), 73.3 (C-2″), 72.2 (C-3″), 69.9 (C-5‴), 69.9 (C-2‴), 68.3 (C-5″), 67.0 (C-3″), 19.5 (4″-COCH_3_), 19.5 (2″-COCH_3_), 16.7 (C-6‴), 16.2 (C-6″). The above data were identical to the literature data [47].

Kaempferol-3-O-(3-O-acetyl-α-L-rhamnopyranoside) (**21**). Pale yellow amorphous powder, mp 181-183°C. IR (KBr, cm^−1^) *ν*_max_: 3398, 1744, 1603, 994. ^1^H-NMR (CD_3_OD, 500 MHz) δ: 7.70 (2H, d, *J* = 8 Hz, H-2^′^, 6^′^), 6.91 (2H, d, *J* = 5 Hz, H-3^′^, 5^′^), 6.34 (1H, d, *J* = 2 Hz, H-8), 6.20 (1H, d, *J* = 2 Hz, H-6), 5.45 (1H, d, *J* = 2.5 Hz, H-1″), 4.40 (1H, m, H-2″), 2.02 (3H, s, COOCH_3_). ^13^C-NMR (CD_3_OD, 125 MHz) δ: 159.1 (C-2), 135.3 (C-3), 179.1 (C-4), 172.5 ( COCH_3_), 165.2 (C-7), 162.4 (C-4^′^), 160.9 (C-5), 158.1 (C-9), 131.6 (C-2^′^, 6^′^), 122.2 (C-1^′^), 116.3 (C-3^′^, 5^′^), 106.0 (C-10), 102.1 (C-1″), 100.0 (C-6), 95.0 (C-8), 74.7 (C-3″), 71.3 (C-5″), 70.0 (C-4″), 69.0 (C-2″), 21.5 (COCH_3_), 17.7 (C-6″). The above data were identical to the literature data [48].

2α,3α,24-trihydroxyurs-12-en-28-oic acid (**22**). White powder, mp 204-206°C. IR (KBr, cm^−1^) *ν*_max_: 3420-3390, 1690, 1365, 910. ^1^H-NMR (CD_3_OD, 500 MHz) δ: 3.72 (1H, br s, H-3), 2.13 (1H, d, *J* = 5.5Hz, H-18), 1.11 (3H, s, H-25), 1.06 (9H, s, H-24, 26, 27), 0.94 (3H, d, *J* = 5 Hz, H-29), 0.86 (3H, d, *J* = 2 Hz, H-30). ^13^C-NMR (CD_3_OD, 125 MHz) δ: 179.7 (C-28), 138.5 (C-13), 125.3 (C-12), 73.2 (C-3), 65.6 (C-24), 64.5 (C-2), 53.1 (C-18), 44.1 (C-4), 41.4 (C-14), 41.3 (C-1), 39.5 (C-19), 39.1 (C-20), 39.1 (C-10), 39.1 (C-8), 37.8 (C-22), 33.2 (C-7), 29.4 (C-21), 27.8 (C-15), 24.0 (C-16), 23.2 (C-11), 22.8 (C-23), 21.9 (C-27), 20.3 (C-30), 17.9 (C-29), 17.9 (C-6), 16.4 (C-26), 16.4 (C-25). The above data were identical to the literature data [49].

Kaempferol-3-O-α-L-rhamnopyranoside-7-O-α-L-rhamopyranoside (**23**). Yellow powder, mp 200-202°C. IR (KBr, cm^−1^) *ν*_max_: 3420, 2911, 1651, 1605, 1022. ^1^H-NMR (CD_3_OD, 500 MHz) δ: 7.78 (2H, d, *J* = 7 Hz, H-2^′^, -6^′^), 6.92 (2H, d, *J* = 5.5 Hz, H-3^′^, -5^′^), 6.71(1H, d, *J* = 1.5 Hz, H-8), 6.44 (1H, d, *J* = 1.5 Hz, H-6), 5.54 (1H, br s, H-1″), 5.38 (1H, s, 2‴-OH), 4.57 (1H, br s, H-2″), 4.00 (1H, br s, H-2‴), 3.80 (1H, m, H-3″), 3.59 (1H, m, H-3‴). ^13^C-NMR (CD_3_OD, 125 MHz) δ: 178.4 (C-4), 162.2 (C-7), 161.6 (C-5), 160.4 (C-4^′^), 158.4 (C-2), 156.7 (C-9), 135.1 (C-3), 130.6 (C-3^′^,-5^′^), 121.0 (C-1^′^), 115.2 (C-2^′^, -6^′^), 106.2 (C-10), 102.1 (C-1″), 99.2 (C-6), 98.5 (C-1‴), 94.2 (C-8), 72.3 (C-2″), 71.8 (C-4‴), 70.8 (C-4″), 70.7 (C-3‴), 70.7 (C-5‴), 70.5 (C-3″), 70.3 (C-2‴), 69.9 (C-5″), 16.7 (C-6‴), 16.4 (C-6″). The above data were identical to the literature data [50].

### Cell lines and culture

MGC-803, PC3, A375, and NIH3T3 cell lines were obtained from the Institute of Biochemistry and Cell Biology, China Academy of Science. MGC-803 is stomach cancer cell line, PC3 is prostate cancer cell line, A375 is malignant melanoma cell line, and NIH3T3 is mouse fibroblast cell line. The entire cancer cell lines were maintained in the RPMI 1640 medium and NIH3T3 was maintained in the DMEM medium. They were supplemented with 10% heat-inactivated fetal bovine serum (FBS) in a humidified atmosphere of 5% CO_2_ at 37°C. All cell lines were maintained at 37°C in a humidified 5% carbon dioxide and 95% air incubator.

### MTT assay

All tested extracts and compounds were dissolved in DMSO and subsequently diluted in the culture medium before treatment of the cultured cells. When the cells were 80-90% confluent, they were harvested by treatment with a solution containing 0.25% trypsin, thoroughly washed and resuspended in supplemented growth medium. Cells (2 × 10^3^/well) were plated in 100 μL of medium/well in 96-well plate. After incubations overnight, the cells were treated with different concentrations of extracts in RPMI 1640 with 10% FBS for 72 h. In parallel, the cells treated with 0.1% DMSO served as negative control and ADM (Adriamycin) as positive control. Finally, 100 μL of MTT (Beyotime Co., Jiangsu, China) was added, and the cells were incubated for 4 h. The MTT-formazan formed by metabolically viable cells was dissolved in 100 μL of SDS for 12 h. The absorbance was then measured at 595 nm with a microplate reader (BIO-RAD, model 680), which is directly proportional to the number of living cells in culture [51].

### AO/EB staining

The cells were seeded at a concentration of 5 × 10^4^ cell/mL in a volume of 0.6 mL on a sterile cover slip in 6-well tissue culture plates. Following incubation, the medium was removed and replaced with fresh medium plus 10% FBS and then supplemented with compounds. After the treatment period, the cover slip with monolayer cells was inverted on the glass slide with 20 μL of AO/EB (Beyotime Co., Shanghai, China) stain (100 μg/mL). The fluorescence was read using an IX71SIF-3 fluorescence microscope (OLYMPUS Co., Toukyu Met, Japan).

### Hoechst 33258 staining

The cells grown on the sterile cover slip in 6-well tissue culture plates were treated with compounds for a certain range of treatment time. The culture medium containing compounds was removed, and the cells were fixed in 4% paraformaldehyde for 10 min. The cells were washed twice with PBS, and were consequently stained with 0.5 mL of Hoechst 33258 staining (Beyotime Co., Jiangsu, China) for 5 min. The stained nuclei were washed twice with PBS, and were consequently observed under an IX71SIF-3 fluorescence microscope at 350 nm excitation and 460 nm emissions.

### TUNEL assay

TUNEL assays were performed using a colorimetric TUNEL apoptosis assay kit according to the manufacturer’s instructions (Beyotime). The cells grown in 6-well culture clusters were treated as mentioned in mitochondrial depolarization assay. The MGC-803 cells grown in 6-well tissue culture plates were washed with PBS and fixed in 4% paraformaldehyde for 40 min. The cells were washed once with PBS, and were consequently permeabilized with immunol staining wash buffer (Beyotime) for 2 min on ice. The cells were rewashed once with PBS, and were consequently incubated in 0.3% H_2_O_2_ in methanol at room temperature for 20 min to inactivate the endogenous peroxidases, after which the cells were washed thrice with PBS. Thereafter, the cells were incubated with 2 μL of TdT-enzyme and 48 μL of Biotin-dUTP per specimen for 60 min at 37°C. The cells were terminated for 10 min, and were consequently incubated with streptavidin-HRP (50 μL per specimen) conjugate diluted at 1:50 in sample diluent for 30 min. The cells were washed three times with PBS, and were consequently incubated with diaminobenzidine solution (200 μL per specimen) for 10 min. Thereafter, the cells were rewashed twice with PBS, and were consequently imaged under an XDS-1B inverted biological microscope (Chongqing Photoelectric Devices Co. Chongqing, China).

### Flow cytometry analysis

Prepared MGC-803 cells (1 × 10^6^/mL) were washed twice with cold PBS and then re-suspended gently in 500 μL binding buffer. Thereafter, cells were stained in 5 μL Annexin V-FITC and shaked well. Finally, 5 μL PI was added to these cells and incubated for 20 min in a dark place, analyzed by FACS Calibur, Becton Dickinson.

### Statistical analysis

All statistical analyses were performed using SPSS 10.0, and the data were analyzed using one-way ANOVA. The mean separations were performed using the least significant difference method. Each experiment was performed in triplicate, and all experiments were run thrice and yielded similar results. Measurements from all the replicates were combined, and the treatment effects were analyzed.

## Competing interests

All the authors declare that they have no competing interests.

## Authors’ contributions

Shengjie Yang and Mingchuan Liu performed the experiments, analyzed the data and wrote the paper. Na Liang and Qi Zhao performed the experiments. Yuping Zhang and Wei Xue planned and analyzed the data, and Song Yang planned the experiments, wrote the paper and give final approval of the version to be published. All authors contributed to this study, read and approved the final manuscript.
